# Polyethylene Terephthalate and Endocrine Disruptors

**DOI:** 10.1289/ehp.1001986

**Published:** 2010-05

**Authors:** Ralph Vasami

**Affiliations:** PETRA (PET Resin Association), New York, New York, E-mail: rvasami@PETresin.org

In the commentary “Polyethylene Terephthalate May Yield Endocrine Disruptors,” Sax (2010) theorized that bottles made of polyethylene terephthalate (PET) might leach phthalate ester plasticizers and/or antimony to produce endocrine-disrupting effects. On behalf of the North American producers of PET resin, I have the following comments and corrections.

Phthalate ester plasticizers are not used to manufacture polyethylene terephthalate and never have been. It is not chemically plausible for PET to produce these phthalate esters.

Sax (2010) did suggest that some reports of phthalate esters in PET bottled water containers may have originated from contamination of the bottled water, or from phthalate ester contamination of recycled PET used in manufacturing water and beverage containers. In addition, non-PET components of bottled water containers (e.g., closures) might be another possible source. Whatever the origin of phthalate esters, which could not be identified in any of the studies cited by Sax, it is clearly unreasonable to ascribe PET as the source.

Regarding antimony, Sax noted that Choe et al. (2003) reported antimony chloride as showing high estrogenicity. However, antimony oxides—not antimony chloride—are used as catalysts in the manufacture of PET. Antimony oxides are chemically and toxicologically distinct from antimony chlorides. No study has reported finding toxic amounts of antimony in PET-bottled water or beverages.

PET bottles and containers meet all applicable U.S. and international safety requirements for food contact, and the inert qualities of PET define its preferred use for many food, beverage, and medical applications. Consumers can feel confident about the safety of PET food and beverage containers.

We welcome dialogue with researchers and regulatory agencies on the chemistry and safety of PET resin.
